# Prevalence and Evolution of Low Frequency HIV Drug Resistance Mutations Detected by Ultra Deep Sequencing in Patients Experiencing First Line Antiretroviral Therapy Failure

**DOI:** 10.1371/journal.pone.0086771

**Published:** 2014-01-27

**Authors:** Marie-Anne Vandenhende, Pantxika Bellecave, Patricia Recordon-Pinson, Sandrine Reigadas, Yannick Bidet, Mathias Bruyand, Fabrice Bonnet, Estibaliz Lazaro, Didier Neau, Hervé Fleury, François Dabis, Philippe Morlat, Bernard Masquelier

**Affiliations:** 1 Université Bordeaux, Microbiologie fondamentale et Pathogé nicité, UMR 5234, Bordeaux, France; 2 Centre national de la recherche scientifique, Microbiologie fondamentale et Pathogé nicité, UMR 5234, Bordeaux, France; 3 Centre Hospitalier Universitaire de Bordeaux, Laboratoire de virologie, Bordeaux, France; 4 Centre Hospitalier Universitaire de Bordeaux, Service de Médecine Interne et Maladies Infectieuses, Hôpital Saint-André, Bordeaux, France; 5 Centre Jean Perrin, Plateforme GINA, EA 4677 ERTICa, Clermont-Ferrand, France; 6 Université Bordeaux, ISPED, Centre Inserm U897-Epidémiologie-Biostatistique, Bordeaux, France; 7 Centre Hospitalier Universitaire de Bordeaux, COREVIH Aquitaine, Bordeaux, France; 8 Institut National de la Santé et de la Recherche Mé dicale, ISPED, Centre Inserm U897-Epidémiologie-Biostatistique, Bordeaux, France; 9 Centre Hospitalier Universitaire de Bordeaux, Service de Médecine Interne et Maladies Infectieuses, Hôpital Haut-Lévêque, Bordeaux, France; 10 Centre Hospitalier Universitaire de Bordeaux, Fédération de maladies infectieuses et tropicales, Hôpital Pellegrin, Bordeaux, France; Centro de Biología Molecular Severo Ochoa (CSIC-UAM), Spain

## Abstract

**Objectives:**

Clinical relevance of low-frequency HIV-1 variants carrying drug resistance associated mutations (DRMs) is still unclear. We aimed to study the prevalence of low-frequency DRMs, detected by Ultra-Deep Sequencing (UDS) before antiretroviral therapy (ART) and at virological failure (VF), in HIV-1 infected patients experiencing VF on first-line ART.

**Methods:**

Twenty-nine ART-naive patients followed up in the ANRS-CO3 Aquitaine Cohort, having initiated ART between 2000 and 2009 and experiencing VF (2 plasma viral loads (VL) >500 copies/ml or one VL >1000 copies/ml) were included. Reverse transcriptase and protease DRMs were identified using Sanger sequencing (SS) and UDS at baseline (before ART initiation) and VF.

**Results:**

Additional low-frequency variants with PI-, NNRTI- and NRTI-DRMs were found by UDS at baseline and VF, significantly increasing the number of detected DRMs by 1.35 fold (p<0.0001) compared to SS. These low-frequency DRMs modified ARV susceptibility predictions to the prescribed treatment for 1 patient at baseline, in whom low-frequency DRM was found at high frequency at VF, and 6 patients at VF. DRMs found at VF were rarely detected as low-frequency DRMs prior to treatment. The rare low-frequency NNRTI- and NRTI-DRMs detected at baseline that correlated with the prescribed treatment were most often found at high-frequency at VF.

**Conclusion:**

Low frequency DRMs detected before ART initiation and at VF in patients experiencing VF on first-line ART can increase the overall burden of resistance to PI, NRTI and NNRTI.

## Introduction

The advent of combination antiretroviral therapy (ART) has dramatically reduced HIV-1 infection-related morbidity and mortality [1]. However, the efficiency of these treatments can be compromised by the presence of drug-resistant variants, resulting in virological failure [2]. According to epidemiological studies, 8–11% of antiretroviral naive patients are infected with a virus harbouring drug resistance associated mutations (DRMs) in Europe and the USA [3]. Treatment guidelines therefore recommend genotypic resistance testing before initiating antiretroviral therapy and in the case of virological failure [4].

Standard genotyping by Sanger sequencing (SS) used currently in clinical practice cannot detect viral variants representing less than 15–25% of the viral population [5]. More sensitive techniques have been developed, including ultra-deep sequencing (UDS), which can detect and quantify low-frequency variants harbouring DRMs down to 0.5–1% [6].

Clinical relevance of detecting low-frequency DRMs remains open to debate. Some studies have found no significant association between the presence of low-frequency DRMs and subsequent virological failure [7]–[9] while others reported an overt correlation [10]–[14]. A recent pooled analysis showed that low-frequency non-nucleoside reverse transcriptase inhibitor (NNRTI)-DRMs increased the risk of virological failure (VF) with NNRTI-based regimen more than two-fold [15]. The impact of low-frequency protease inhibitor (PI)-DRMs on treatment response has been limited to a few studies that found no associations [11], [16].

The objectives of our study were to determine the prevalence of DRMs detected by UDS as well as their effect on ART resistance before treatment and at VF, and to analyse their evolution under treatment, in HIV-1 infected patients experiencing VF on first-line ART.

## Methods

### Ethics statement

All patients included in this study gave written informed consent. The study protocol was approved by the Ethics committee of Bordeaux University Hospital (Comité de protection des personnes).

The Agence Nationale de Recherche sur le SIDA (ANRS) CO3 Aquitaine Cohort is a prospective hospital-based cohort of HIV-1 infected patients under routine clinical management, initiated in 1987 in the Bordeaux University Hospital and four other public hospitals in the Aquitaine region, South Western France. Inclusion criteria are: adult patients of the participating hospital wards with confirmed HIV-1 infection, having at least one follow-up after the first report, and having given informed consent. Visits occur usually every three months if the patient is treated, every six months otherwise. Detailed presentation of the cohort has been reported elsewhere [17].

### Study population

Patients starting a first antiretroviral treatment between January 2000 and June 2009 were retrospectively screened from the ANRS CO3 Aquitaine Cohort database.

Patients experiencing virological failure (VF), defined as a plasma viral load (VL) >1,000 copies/ml or 2 consecutives VL>500 cp/ml at least 6 months after ART initiation, and with plasma samples available both at baseline (last sample available before ART initiation) and at VF (plasma sample corresponding to first VL>1000 or second VL>500 cp/ml) were included in our study. Patients were excluded if they changed or stopped their ART. Socio-demographic, clinical and biological characteristics of eligible participants were extracted from the database.

### Sanger sequencing

Viral RNA was extracted from 1 ml of plasma sample at baseline and at VF using the High Pure Viral RNA kit (Roche). The Reverse Transcriptase (RT) and Protease gene sequences were determined using Sanger sequencing (SS) according to the ANRS consensus method (http://www.hivfrenchresistance.org).

### Ultra-deep sequencing (UDS)

Viral RNA was extracted as described above and used as a template for RT-PCR. The *Pol* region (HxB2 position: 2082–3420) was amplified using the 5P1 and MJ4 primers and the SuperScript III One-Step RT-PCR System with Platinum Taq DNA Polymerase kit (Invitrogen, Carlsbad, CA). RT-PCR conditions were reverse-transcription at 50°C for 30 minutes, one denaturation step of 94°C for 2 minutes, followed by 40 cycles of denaturation for 30 seconds at 94°C, annealing for 30 seconds at 55°C, extension for 90 seconds at 68°C, and a final 7-minutes extension at 68°C.

Two microliters of RT-PCR product were used to generate 4 amplicons (Prot, RT1, RT2 and RT3) encompassing protease and RT regions and harbouring specific MIDs in their end. Amplicons and primers are described in **Table 1**. The nested PCR was performed with the FastStart High Fidelity PCR System (Roche Diagnosis) and according to the following conditions: initial denaturation step of 94°C for 3 minutes followed by 40 cycles of denaturation for 30 seconds at 94°C, annealing for 45 seconds at 55°C, and extension for 60 seconds and a final 8-minutes extension at 72°C.

**Table 1:** Primers used for amplicon generation.

Amplicon	HxB2 position	HIV specific primer (5′-3′)
Prot	2136–2164	TCAGAGCAGACCAGAGCCAACAGCCCCA
	2621–2650	AATGCTTTTATTTTTTCTTCTGTCAATGGC
RT1^1^		-
		-
RT2	2706–2734	GAAAATCCATACAATACTCCAGTATTTGC
	3119–3145	CTATGCTGCCCTATTTCTAAGTCAGAT
RT3^2^	2874–2891	CTRGATGTGGGTGATGCA
	3265–3284	CNYTATAGGCTGTACTGTCC

Note that forward and reverse primers are linked to primer A and B (454 Life Sciences; Roche) respectively and contain the TCAG key. To distinguish each sample in the multiplexed UDPS, nine unique sequence tags (MID1 to 9, according Roche's protocol) were inserted between the adaptor A or B and the gene specific primer.

1 RT1: primers under patent process

2 Primers from Mitzuya's paper [29]

PCR products were purified using magnetic beads (Agencourt AMPure Kit, Beckman Coulter, and Benried, Germany) to eliminate primer-dimers. The number of molecules was quantified by fluorometry (Quant-iT PicoGreen dsDNA assay kit, Invitrogen, Carlsbad, CA) and equimolar concentrations of amplicons were pooled. This library was submitted to emulsion-based clonal amplification (emPCR) using GS Junior Titanium emPCR Kit (Lib-A) according to Roche's protocol. UDS was performed in both strands on a 454 Life Science Roche GS junior. Amplicon Variant Analyzer (AVA 2.7) software (454 Life Sciences; Roche) was used to align all read amplicons and to calculate variant frequencies at each nucleotide position relative to the HxB2 reference HIV-1 strain sequence. Raw data were submitted to GenBank under SRP033482 study accession number.

### UDS technical error rate

To estimate the error rate of our UDS protocol, we calculated the percentage of errors generated from 2 steps of PCR (Platinum Taq Polymerase followed by FastStart High Fidelity polymerase amplification) and pyrosequencing of a plasmid control containing HIV-1 LAV reference HIV-1 strain (pNL4.3). The mean error rate was calculated using a triplicate experiment and the percentage of variations compared to the plasmid HIV-1 sequence. We estimated an overall mean error rate of 0.21±0.07% (mean reads per position: 3007±1477). Deletion and insertions events corresponded to 29.8% and 61.9% of the overall errors, with 0.07±0.06% of deletion and 0.12±0.07% of insertion relative to total reads. These values are in accordance with the rate of errors generated by the FastStart High Fidelity polymerase [18].

As input HIV RNA was not quantified, the expected sampling error depended on sample viral load. The stochastic effects of sampling variation were limited when the starting RNA copy number was higher than 10,000 copies/mL [6]. Below this threshold, the proportion of sequenced PCR amplicons containing DRMs may not be representative to the original sample.

Drug resistant mutations (DRMs) were thus accepted as significant variants when present at a frequency of >1% among the total number of reads and in both strands.

### Drug resistance mutations and Genotypic Sensitivity Score

Reverse transcriptase and protease DRMs were identified using SS and UDS at baseline and VF. Analyzed mutations associated with resistance to nucleoside reverse transcriptase inhibitor (NRTI), NNRTI and PI were referenced in the 2011 International AIDS Society (IAS)–USA drug resistance mutations list [19]. Genotypic resistance was interpreted with the 2012 ANRS HIV drug resistance algorithm v22 (http://www.hivfrenchresistance.org) in order to determine the overall burden of resistance to antiretroviral (ARV) drugs and resistance to the prescribed treatment. The weighted Genotypic Sensitivity Score (wGSS) scores the resistance to the prescribed treatment and was calculated as the sum of the scores obtained for each prescribed ARV drug. In this system, ARV drugs are considered to display full, intermediate or null antiviral activity and scored as 1, 0.5 or 0 for PIs, 1, 0, 0 for NNRTIs and 0.5, 0, 0 for NRTIs [20].

### Statistical tests

Paired t-tests were used to compare the mean number of DRMs detected by SS versus UDS for all DRMs, PI-DRMs, NRTI-DRMS and NNRTI-DRMs (Stata 12.1 software).

## Results

### Study population

Twenty-nine patients achieving VF at least 6 months after initiating first-line ART had plasma samples available at baseline and at VF and were included in our study (**Table 2**). They were mainly men (79.3%) and infected with subtype B HIV-1 in 72.4% of the cases. Median baseline CD4 cell counts (cells/mm3) and median VL (log_10_ copies/ml) were 248 (interquartile range IQR: 177–314) and 5.0 (IQR: 4.6–5.2) respectively. After a median duration of 14.3 months under therapy (IQR: 8.7–24.2), VF occurred in patients with a median of 3.7 log_10_ copies/ml of HIV RNA (IQR: 3.3–4.0).

**Table 2:** Demographic, clinical and virological characteristics of the included patients (n = 29).

Patients characteristics		n (%)
Gender male, n (%)		23 (79.3%)
Median age, years (IQR)		36.8 (33.0–39.4)
Transmission group, n (%)	MSM	13 (44.8%)
	IDU	5 (17.3%)
	Heterosexual	9 (31.0%)
	Other	2 (6.9%)
HIV-1 B subtype, n (%)		21 (72.4%)
Antiretroviral treatment, n (%)	2 NRTIs	3 (10.3%)
	3 NRTIs	5 (17.2%)
	2 NRTI + PI	3 (10.3%)
	3 NRTI + PI	2 (6.9%)
	2 NRTI + PI/r	8 (27.6%)
	3 NRTI + PI/r	2 (6.9%)
	2 NRTI + NNRTI	6 (20.8%)
Period of ART initiation	<2003	17 (58.6%)
	≥2003	12 (41.4%)
Median baseline plasma HIV-1 RNA, log_10_ copies/ml (IQR)		5.0 (4.6–5.2)
Median baseline CD4 cell count, cells/mm^3^ (IQR)		248 (177–314)
Median VF plasma HIV-1 RNA, log_10_ copies/ml (IQR)		3.7 (3.3–4.0)
Median time between baseline and VF, month (IQR)		14.3 (8.7–24.2)

MSM: men who have sex with men, IDU: intravenous drug user, IQR: interquartile range, VF: virological failure

Three patients were treated with a 2 NRTI-based regimen, 5 patients with 3 NRTIs, 5 patients with NRTIs + PI, 10 patients with NRTIs + boosted PI and 6 patients with 2 NRTIs + NNRTI. Co-prescribed PIs were Atazanavir, Lopinavir, Nelfinavir or Fosamprenavir and co-prescribed NNRTIs were Efavirenz or Nevirapine.

### Sequencing

Sequencing was performed at baseline on the last plasma sample available before ART initiation (median time period between the baseline sample and the start of ART: 28 days, IQR: 8–91) and on the plasma sample corresponding to the date of VF. UDS performed on baseline viral HIV RNA yielded a median of 1663 (IQR: 1017–2533) reads per amplicon (VL range: 3.08 log_10_ cp/ml to 6.62 log_10_ cp/ml), and a median of 1739 (IQR: 1123–2585) reads per amplicon on viral HIV RNA from plasma collected at VF (VL range: 3.01 log_10_ cp/ml to 4.98 log_10_ cp/ml at failure). UDS of the protease region was successful for all of the 29 patients at failure and at baseline but RT UDS failed for 1 patient at baseline.

**Figure 1:**
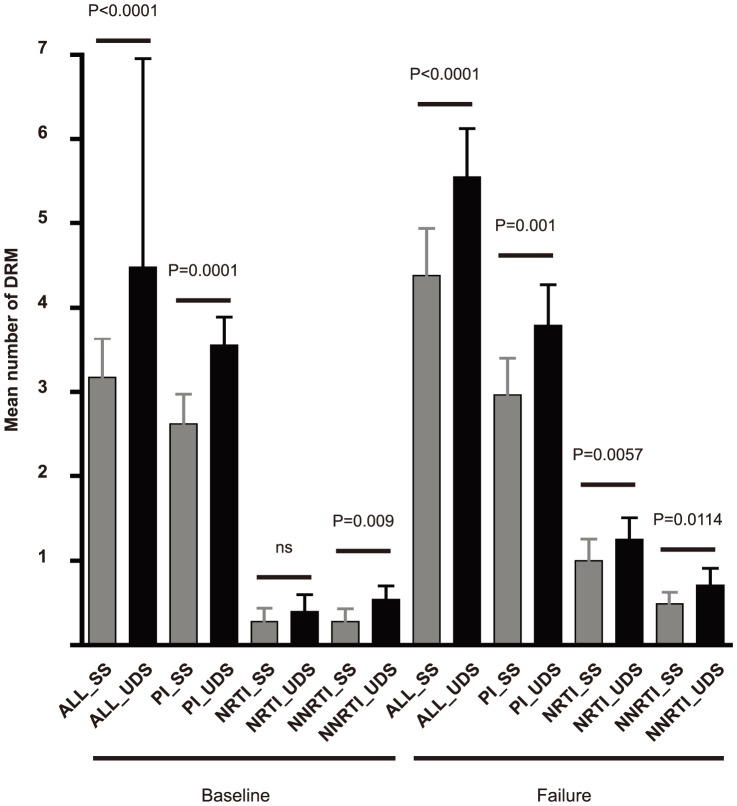
Drug resistant mutations (DRMs) detected by Sanger sequencing (SS) and ultra-deep sequencing (UDS). Mean of DRMs per patients were obtained. Two-tailed p values and 95% confidence intervals were calculated from the paired Student t-test (ns: not statistically significant, p>0.05). ALL_SS and ALL_UDS: all DRMs detected by SS and UDS respectively PI_SS and PI_UDS: Protease inhibitor (PI)-DRMs detected by SS and UDS respectively NRTI_SS and NRTI_UDS: Nucleoside Reverse Transcriptase Inhibitor (NRTI)-DRMs detected by SS and UDS respectively. NNRTI_SS and NNRTI_UDS: Non-Nucleoside Reverse Transcriptase Inhibitor (NNRTI)-DRMs detected by SS and UDS respectively.

Comparison of the mean number of DRMs detected by the 2 sequencing techniques (**Figure 1**) revealed that 1.35-fold more DRMs were detected by UDS (1.4-fold more at baseline and 1.3 at VF, p<0.0001). All the DRMs found by SS were also detected by UDS. Additional low-frequency DRMs were found in 21/29 patients at baseline and in 18/29 patients at failure. The difference between the mean number of DRMs detected by SS and by UDS was statistically significant (p<0.05) at baseline for PI and NNRTI-DRMs and at VF for the 3 antiviral drug classes.

### Baseline mutations (Tables 3 and 4)

**Table 3:** List of drug resistance mutations (DRM) detected at baseline (BL) and virological failure (VF) by Sanger sequencing (SS) and UDS for patients failing a PI-based regimen (n = 15).

Pt.	BL VL (cp/ml)	Baseline DRM SS and *UDS (%)*			BL GSS		ART	VF VL (cp/ml)	VF DRM SS and *UDS (%)*			VF GSS	
		Protease	NRTI	NNRTI	SS	UDS			Protease	NRTI	NNRTI	SS	UDS
4	125416	L10I, M36I, D60E, L63P, A71T, **G48V**	M41L, D67N, L210W, T215Y	V179T, *E138G (2.1)*	2	2	ZDV 3TC DDI NFV	5914	L10I, M36I, D60E, L63P, A71T, **G48V**, **V82A**	M41L, D67N, L210W, T215Y, M184V	V179T	0.5	0.5
14	1292950	M36I, I62V, L63P, A71T, V77I	0	0	2.5	2.5	ZDV 3TC ABC NFV	8435	M36I, I62V, L63P, A71T, V77I	*M184I (4.3)*	*Y188C (2.0)*	2.5	2
16	20103	V77I, *L63P (1.5)*	0	0	2.5	2.5	ZDV 3TC ABC LPV/r	1170	V77I	0	0	2.5	2.5
23	121221	*M36I (13.1), A71T (11.2)*	0	0	2	2	ZDV 3TC NFV	2414	*M36I (15.4)*	0	0	2	2
25	119112	G16E, I62V, L63P, V77I	L210W, T215D, *M41L* * (4.0)*	0	2	2	3TC TDF LPV/r	97597	G16E, I62V, L63P, V77I, *D60E (1.5)*	L210W, T215D, M41L	0	2	2
31	198233	L10V, G16E, K20I, M36I, H69K, L89M	0	0	2	2	ZDV 3TC LPV/r	59663	L10V, G16E, K20I, M36I, H69K, L89M	0	0	2	2
40	96878	K20R, M36I, I62V, L63P, *M36L (2.1)*	0	0	2	2	ZDV 3TC LPV/r	2021	K20R, M36I, I62V, L63P,**M46L**,**I54V** **, V82A**,*L10V* * (5.0)*	M184V	0	1.5	0.5
41	771	K20I, M36I, H69K, L89M	0	0	2	2	3TC TDF ATZ/r	1530	K20I, M36I, H69K, L89M	0	0	2	2
43	83525	K20I, M36I, H69K, L89M	0	0	2	2	ZDV 3TC NFV	9819	K20I, M36I, H69K, L89M, D60E, L63P, **I54V,** **L90M**, *L89I (26.3)*, ***M46L (5.0)***	M184V	0	0.5	0.5
46	140551	*M36I (1.0)*	0	0	2.5	2.5	ZDV 3TC ABC LPV/r	14505	M36I, *V11I (1.5)*	*L74V (3.0)*	0	2.5	2
49	232736	K20R, V77I, *M36I (32.6), L63P (3.4)*	0	V90I	2	2	3TC ABC FPV/r	2387	V77I	*M184I (2.7)*	V90I	*2*	*1.5*
51	19200	G16E, K20R, M36I, *I62V (8.5), V77I (4.6)*	0	0	2	2	TDF FTC LPV/r	12796	G16E, K20R, M36I, *V77I (1.9)*	M184V, *M184I (2.2)*	*K101E (6.8)*	*1.5*	*1.5*
52	782200	I62V, L63P, V77I, *M36I (1.3)*	0	*V90I (4.5)*	2	2	TDF FTC ATZ/r	2155	I62V, L63P, V77I, **N88S**,***M46L*** *** (18.8)*** *,_* ***M46I *** ***(8.3)*** *, A71V (9.2), G73S (24.1)*	M184V	0	0.5	0.5
57	142700	M36I, I62V, L63P, A71T, V77I	0	0	2	2	ZDV 3TC LPV/r	4861	M36I, I62V, L63P, A71T, V77I, *G16E (1.6)*	M184V	0	1.5	1.5
79	1108000	M36I, L63P, *G16E (19.3), K20T (5.1), I62V (5.8), V77I (4.4)*	T215D	V90I	2	2	DDI D4T NFV	1693	M36I, L63P, **D30N**	D67N, T215D, M184V	V90I	0.5	0.5

*Italic*: mutations only detected by UDS/Normal: mutations detected by both UDS and SS

**Bold**: Major protease mutations

Underlined: mutations associated to prescribed treatment

GSS, Genotypic Sensitivity Score; ART, antiretroviral treatment; ZDV, zidovudine; DDI, didanosine; D4T, stavudine; 3TC, lamivudine; FTC: Emtricitabine; ABC, abacavir; TDF, tenofovir; NFV, nelfinavir; LPV/r, boosted lopinavir; ATZ/r, boosted atazanavir, FPV/r, boosted fosamprenavir

**Table 4:** List of drug resistance mutations (DRM) detected at baseline (BL) and virological failure (VF) by Sanger sequencing (SS) and UDS for patients failing a NNRTI-based regimen (n = 6) or NRTI-based regimen (n = 8).

Pt.	BL VL (cp/ml)	Baseline DRM SS and *UDS (%)*			BL GSS		ART	VF VL (cp/ml)	VF DRM SS and *UDS (%)*			VF GSS	
		Protease	NRTI	NNRTI	SS	UDS			Protease	NRTI	NNRTI	SS	UDS
20	45028	L63P, *L33V (6.4), M36I (24.8)*	0	0	2	2	ZDV 3TC NVP	6572	L63P, *M36I (6.9)*	M184V	K103N, H221Y, M230L, *V90I (14.5)*	0.5	0.5
28	49268	0	0	0	1.5	1.5	ZDV 3TC ABC	88025	*G16E (2.7), L33V (2.6), M36I (1.8), V77I (2.0)*	0	0	1.5	1.5
38	38779	M36I	0	0	1.5	1.5	ZDV 3TC ABC	9132	0	M184V	0	1	1
47	1324	K20I, M36I, H69K, L89M	0	0	2	2	3TC FTC EFV	2160	K20I, M36I, H69K, L89M	0	0	2	2
61	33676	K20I, M36I, H69K, L89M, *G16E (1.1)*	0	*Y181C (2.1)*	2	1	3TC D4T NVP	533	K20I, M36I, H69K, L89M, *H69R (4.4), * ***D30N (1.3)***	M184I	Y181C, V90I	0.5	0.5
62	67288	L63P, *D60E (8.5), I62V (3.0), V77I (12.0)*	0#	V90I#	2	2	ZDV 3TC NVP	10371	I62V, *L33V (2.9), L63P (35.3)*	0	Y181C, V90I,*K101E* * (4.2)*	1	1
63	88509	G16E, M36I, I62V, H69K, *K20R (11.6)*	ND	ND	ND	ND	ZDV 3TC ABC	676	G16E, M36I, I62V, H69K K20R	M184V *	0*	1	ND
64	141586	V11I, K20I, M36I, H69K, L89M, *V77I (6.7)*	0#	0#	2	2	3TC TDF EFV	41115	V11I, K20I, M36I, H69K, L89M	*M184V (3.8)*	G190S, *V90I (45.0)*	1	0.5
76	15857	I62V, *M36I (2.7), * ***Q58E (2.5)*** *, L63P (2.3)*	0	*V106I (11.0), V90I (1.9)*	1	1	ZDV 3TC	2438	I62V	M184V, *K70R* * (15.2)*	0	0.5	0,5
80	1607	D60E, L63P	D67N, K70R, T215Y, K219E, *M41L (2.6)*	0	0	0	DDI D4T	2033	D60E, L63P	D67N, K70R, T215Y, K219E, M41L	0	0	0
82	586670	L63P, V77I	*L210W (9.0)*	K103N	1.5	1.5	ZDV 3TC ABC	2667	L63P, V77I, *D60E (2.8)*	M184V	K103N	1	1
83	6741	L63P, *K20R (4.0), M36I (7.5)*	0	*V90I (6.6)*	1	1	ZDV 3TC	1812	L63P	M184V	0	0.5	0.5
85	147650	0	0	*V90I (2.2)*	2	2	3TC TDF EFV	3306	0	0	V90I	2	2
86	57839	V77I	0	*E138G (1.1)*	1.5	1.5	ZDV 3TC ABC	6540	0	M184V,*Y115F* * (34.9)*	V90I, *H221Y (8.6)*	1	0.5

* UDS failed for this sample. Only DRMs detected by SS were listed.

# For technical reasons, E138 position was not read by UDS.

*Italic*: mutations only detected by UDPS/Normal: mutations detected by both UDPS and SS.

**Bold**: Major protease mutations.

Underlined: mutations associated to prescribed treatment.

GSS, Genotypic Sensitivity Score; ART, antiretroviral treatment; ZDV, zidovudine; DDI, didanosine; D4T, stavudine; 3TC, lamivudine; FTC: Emtricitabine; ABC, abacavir; TDF, tenofovir; EFV, efavirenz; NVP, nevirapine.

#### Prevalence of DRMs

Using Sanger sequencing, PI mutations were detected in most of the patients but they were accessory or polymorphic IAS-USA mutations except for Patient 4 who had virus harbouring a major PI mutation. NRTI-DRMs were found in 13.8% (4/29) of the patients and NNRTI-DRMs in 17.2% (5/29). UDS analysis showed that 51.7% (15/29), 3.4% (1/29), 10.7% (3/28) and 25.0% (7/28) of the patients had viruses harbouring additional low-frequency PI-, major PI-, NRTI- and NNRTI-DRMs respectively at baseline. The low-frequency NNRTI-DRMs corresponded to polymorphic Etravirine mutations (V90I, V106I) and Rilpivirine mutations (E138G) in a total of 4 and 2 patients respectively.

These low-frequency DRMs could increase the overall burden of resistance to one or more ARV drugs in 4 of 29 patients (13.8%).

#### Association of low-frequency DRMs with the prescribed treatment

Among the 21 patients with low-frequency DRMs, 8 had virus harbouring DRMs correlated with the future prescribed treatment. Nevertheless, the wGSS was not modified except for Patient 61 who harboured 2.1% of HIV variants with Y181C mutation that could imply resistance to its NNRTI treatment.

### VF mutations (Tables 3 and 4)

#### Prevalence of DRMs

Using Sanger sequencing, we determined that 20/29 patients had viruses harbouring DRM. Accessory PI mutations were detected in most of the patients but major-PI mutations were found in 17.2% of patients (5/29). NRTI- and NNRTI-DRMs were found in 58.6% (17/29) and 34.5% (10/29) of the patients respectively.

Additional low-frequency PI-, major PI-, NRTI- and NNRTI-DRMs were found by UDS in 44.8% (13/29), 10.3% (3/29), 25% (7/28) and 21.4% (6/28) of the patients respectively. These low-frequency DRMs could increase the overall burden of resistance to one or more ARV drug in 9 of 29 patients (31%).

#### Association of low-frequency DRMs with the prescribed treatment

Among the 18 patients with low-frequency DRMs, 12 had virus harbouring DRMs correlated with the prescribed treatment. These low-frequency DRMs detected only by UDS led to changes in susceptibility predictions to the prescribed treatment in 6/29 patients (20.7%), as shown by their VF UDS GSS lower than their VF SS GSS. Patients 14, 49 and 64 presented in their viral population 4.3% of M184I, 2.7% and 3.8% of M184V respectively that could be involved in resistance to the prescribed Lamivudine. Abacavir resistance was identified in Patient 46 who harboured 3% of viruses with L74V mutation and in Patient 86 who was infected with 34.9% of viruses carrying Y115F mutation. In Patient 40, 5 high-frequency PI-DRMs were found, leading to possible resistance to Lopinavir. The detection of 5% of variants with the additional Lopinavir resistance mutation L10V could explain an increase on resistance to this PI.

Taking into account both high-frequency and low-frequency DRMs, 8 patients (Patients 16, 23 25, 28, 31, 41, 47 and 85) had no resistance to their prescribed treatment, and 4 had even no DRMs related to their prescribed treatment (Pt 16, 28, 47 and 85)

### Evolution of the low-frequency DRMs between baseline and virological failure

Among the 38 additional low-frequency DRMs detected at baseline, 9 were related to the future prescribed treatment (8 patients). Among them, 1/5 PI-DRMs (Patient 23), 2/3 NRTI-DRMs (Patients 25 and 80) and 1/1 NNRTI-DRMs (Patient 61) were also detected at VF. All the PI-DRMs were accessory PI-DRMs. In the unique subject with baseline low-frequency NNRTI-DRM that could lead to resistance to the prescribed ART (Patient 61, Y181C at 2.1%), both UDS and SS performed at virological failure showed the evolution of this mutation which was detected at high frequency (97.4%).

Most of the treatment-related mutations found at VF were not detected at baseline by UDS. In 75% of the cases (21/28), VF was associated with the occurrence of NRTI DRMs, mainly the M184I/V mutation. Among the 26 patients receiving Emtricitabine (FTC) or Lamivudine (3TC), viruses harbouring the M184I/V mutation at VF were found in 14 patients by both SS and UDS and in 3 patients by UDS only. However, this M184 mutation was never detected at baseline by UDS. Pre-existing low-frequency TAM-NRTI mutations were found in 2/5 patients from whom high level of TAMs were detected at VF (4% and 2.5% baseline M41L; Patient 25 and 80). Concerning the 4 patients with NNRTI-DRMs at VF, only one (Patient 61) had pre-existing low-frequency NNRTI-DRM at baseline. Among the 5 patients treated with PI-based regimen with viruses harbouring major PI-DRMs related to their treatment, none of these PI-DRMs was detected by UDS at baseline. Most of the polymorphic PI-DRMs were already detected at baseline by both SS and UDS.

## Discussion

Our study gave new insights on the distribution of low-frequency variants harbouring PI-, NRTI- and NNRTI-DRMs present prior to first-line therapy and at virological failure, with emphasis on the evolution of these DRMs under ARV therapy. More than two-thirds of the patients presented additional low-frequency DRMs only detected by UDS, with 1.4-fold more DRMs detected by UDS at baseline and 1.3 fold more at VF. This increased prevalence of DRMs was in concordance with previous reports describing the abundance of low-frequency resistant variants (representing less than 20% of viral population), especially detected by UDS, on treatment-naive patients [10], [11], [16] as well as on treatment-experienced patients [21]–[24], confirming the high sensitivity of this technique for the detection and quantification of DRMs. These low-frequency DRMs detected only by UDS could increase the overall burden of resistance to ARV drugs in 13.8% of the patients at baseline and 31% at VF. However, they led to changes in susceptibility predictions to the prescribed treatment only in 3.5% of the patients at baseline and 20.7% at VF.

Twenty-six patients had a 3TC/FTC backbone in their regimen. The M184I/V mutation was detected in 17 of them at failure (only by UDS in 3 of them). Nevertheless, we never detected this mutation on samples collected prior to treatment even at low frequency. The absence of the M184V mutation in baseline circulating viruses is not in agreement with previous reports describing the detection of low-frequency variants carrying M184V mutations in acute [7], [25] and chronically infected naive patients [9]. One explanation could be the difference of the ultrasensitive assay used. In these studies, allele-specific PCR was performed for M184V detection, with detection threshold lower than our 1% cut-off. Simen *et al* found a very low prevalence of M184V/I by UDS in chronically infected naive patients [11]. We could also hypothesize that this lack of M184I/V-harbouring viruses in treatment-naive patients may be a result of the high fitness cost of the M184V virus, which severely impairs replicative capacity [21]. Longer time periods between seroconversion, and then transmission of putative M184V variants, and plasma collection for baseline UDS in our study could lead, in the absence of selective drug pressure, to the decay of these low-replicative variants to very low levels (under the limit of detection of UDS) or even its elimination from the plasma.

One of the main questions sparked by these new ultrasensitive assays remains the significance and the clinical relevance of these low-frequency DRMs, and especially their role in subsequent virological failure.

In our study, 4/29 patients had viruses harbouring low-frequency NRTI- or NNRTI-DRMs related to the prescribed treatment at baseline and these DRMs were found at high frequency at VF in 3 of them. Moreover, for the unique patient with viruses harbouring low-frequency DRM at baseline (Y181C at 2.1%) which may lead to a resistance to his future treatment by NNRTI, this mutation was found at high frequency (97.4%) by both UDS and SS at virological failure.

The impact of low-frequency DRMs on the subsequent response to ARV likely depends on the percentage of low-frequency resistant variants and/or on the mutational viral load [14], [15]. A recent pooled analysis found an increased risk of VF even at very low NNRTI-DRMs frequencies, but showed a dose-dependent effect of low-frequency DRMs with a significantly higher risk of VF with the presence of low-frequency variant at 1% or greater [15]. In our study, we chose to report only low-frequency DRM present at a threshold greater than 1%, to select the mutations with potentially stronger impact on virological outcome, but also to exclude mutations related to laboratory artefact from reverse transcription, PCR amplification and/or sequencing [18], [26].

The impact on the virological outcome probably also depends on treatment regimen.

Many studies clearly report a strong and significant association between low-frequency NNRTI-DRMs and a higher risk of virological failure in patients treated by NNRTI-based regimen[10]–[15], [27] even with high levels of adherence [28].

The few studies that evaluated the impact of low-frequency PI-DRMs on PI-based regimen found no associations with an increased risk of treatment failure [11], [16]. In our study, PI-DRMs found by UDS in ARV-naive patients were mainly accessory IAS-USA mutations. These accessory PI mutations are polymorphic but their accumulation could impact the susceptibility to some PI such as Lopinavir or Nelfinavir. In addition, none of the major PI-DRMs present at virological failure were detected at low frequency prior to treatment. Nevertheless, major PI-DRMs were present at low frequency at virological failure in 5/15 patients treated with a PI-based regimen. The additional accessory PI-DRMs only detected by UDS at VF led to changes in susceptibility predictions to the prescribed PI in one of the 15 patients and might explain virological failure.

Others studies have shown that low-frequency variants harbouring major PI-mutations are infrequently detected by UDS (most of them are accessory mutations with low Stanford HIVdb scores) and occurred in isolation in ARV-naive patients and in patients experiencing failure on PI boosted-regimen [16], [24]. A study reported that samples with low-frequency PI-DRMs (identified with Stanford-HIVdb weights >12 for Atazanavir and Lopinavir) remained phenotypically susceptible to PIs. This might be explained by the fact that these low-frequency PI-DRMs have been found at levels lower than 0.5–1% [24].

The very low prevalence of variants harbouring major PI-DRMs at significant levels combined with the high genetic barrier to resistance of PI could explain the fact that studies failed to show an impact of low-frequency PI-DRMs on virological response to boosted-PI based regimens. Further studies are needed to determinate if the existence of major PI-DRMs present at low frequency could affect the efficacy of PI/r containing regimens.

Our work has several limitations.

First, the ability to detect low-frequency variants depends on initial VL and on the amount of RNA copies used for RT-PCR. We were not able to quantify the number of RNA copies submitted to amplification procedure. Based on previously published data that consider RNA extraction efficiency, we assumed that the approximate number of templates derived from a sample with plasma HIV-1 RNA levels ≥4.5 log copies/mL and submitted to UDS was around 100–200 copies [6], [29], [30]. The number of RNA templates used for UDS may not be a limiting factor for low-frequency DRMs detection from baseline samples since 76% of them had VL above 4.5 log copies/mL. However, 86% of the failure samples had VL below 4.5 log copies/mL and thus, the probability of finding low-frequency DRMs on these samples may have been then reduced. In addition, the detected low-frequency DRMs could have been over-estimated due to sampling error and selection bias. However, the low-frequency DRMs associated to the treatment detected at failure were in most of the cases found at level >2.5%, increasing the confidence on their reliability.

Secondly, patients were included if they experienced a VF on first-line therapy. The prevalence of low-frequency DRMs on this group of patients was not compared to a control group of patients with virological suppression. Hence, we could not evaluate the impact of the drug resistant low-frequency DRMs on virological outcome. Because of the small number of patients experiencing VF under a first-line ART for whom plasma samples were available both at baseline and VF, only 29 subjects were included in our study. Besides almost 45% of them received an antiretroviral therapy dating back from 2000 which is not state of the art anymore.

Finally, adherence to HAART was not reported. Eight patients had no ARV drug resistance suggesting that their VF was related to non-adherence to treatment. Besides, most of the DRMs found at VF under first-line ARV therapy did not exist prior to treatment and were probably acquired through drug selection pressure, favoured by poor adherence or pharmacokinetic factors. Adherence to antiretroviral therapy is a major predictor of viral suppression and disease progression [31]. Low-frequency NNRTI-DRMs increased the risk of VF across all adherence categories, especially in high level of adherence [28]. Thus this essential parameter has to be taken in consideration for further studies.

In conclusion, UDS identified significantly more DRMs than SS for each class of ARV (NRTI, NNRTI and PI), both in treatment-naive patients and in patients who experienced virological failure on first-line ARV. In few cases, these additional low-frequency DRMs may change the ARV susceptibility predictions to the prescribed treatment.

Large prospective studies are now needed to assess the impact of these low-frequency DRMs on virological response, according to the proportion of each DRM and to the composition of the ARV regimen, before applying these ultrasensitive assays in routine clinical practice.
